# The Influence of Formulation and Excipients on Propranolol Skin Permeation and Retention

**DOI:** 10.1155/2018/1281673

**Published:** 2018-08-02

**Authors:** Cristina Padula, Sara Nicoli, Silvia Pescina, Patrizia Santi

**Affiliations:** Food and Drug Department, University of Parma, Parco Area delle Scienze 27/a, 43124 Parma, Italy

## Abstract

The objective of this work was to study* in vitro* propranolol permeation and skin retention after topical application of different semisolid vehicles, with the final aim of developing new topical formulations intended for the treatment of infantile hemangioma, able to produce therapeutic drug levels in the skin, avoiding systemic absorption. Propranolol ointments, creams, and gels were prepared and tested on pig skin, an accepted model of human skin. From the results obtained in the present work it is clear that the permeation of propranolol across the skin is a poor predictor of its skin retention, at least in the time-frame considered. With an application time of 4 h, reasonably close to the permanence time of a semisolid formulation on the skin surface, the best performance (high retention and low skin penetration) was obtained with lipophilic formulations, in particular with a lipophilic cream containing olive oil. Hydrophilic formulations, such as gels, are characterized by a significant permeation across the skin, probably leading to systemic side effects, accompanied by a limited skin retention. Overall, the results obtained in the present work pose the basis for the development of new topical formulations, containing propranolol, with better performance and reduced systemic absorption.

## 1. Introduction

Infantile hemangioma (IH) is the most common benign tumor of childhood occurring in up to 10% of infants. IH is more common in Caucasians and occurs more frequently in girls and premature infants [1]. Most of IH do not require treatment because of their spontaneous involution. However, a therapeutic intervention could be necessary to avoid functional and aesthetic complications. In 2008 Leaute-Labreze* et al.* [2] accidentally discovered the efficacy of propranolol in treating IH. This finding has revolutionized the therapeutic approach to IH, and propranolol has become the treatment of choice for this pathology, as documented by several studies [3]. Although the precise mechanism of action is not completely known, it affects endothelial cells, vascular tone, angiogenesis, and apoptosis [4]. In 2014, the FDA approved oral propranolol as orphan drug for the treatment of proliferating infantile hemangioma requiring systemic therapy. Oral propranolol has been used in children for decades to treat cardiovascular diseases, so its safety and side effect profile are well known. Nevertheless, there are some concerns since different studies on the efficacy of propranolol in IH reported the occurrence of systemic side effects such hypoglycemia, insomnia, and diarrhea [5].

For superficial or small IH, in which systemic therapy may not be indicated, topical application of propranolol represents a good alternative to oral; this has been reported to be an effective treatment for IH in different* in vivo* studies, recently reviewed by Ovadia [6]. Other beta-blockers, such as timolol, have been investigated for topical use, but propranolol remains the safest alternative; timolol has a potency 4-10 times higher than propranolol and topical application bypasses first-pass liver metabolism, increasing the possibility of systemic side effects [5].

To date no commercial topical form containing propranolol is available on the market and for the* in vivo* studies, galenic formulations have been used. Formulations were prepared by incorporating propranolol hydrochloride (sometimes crushed pills) in petroleum jelly [7–9] or in ointments [10], creams [11, 12], or gels [13–15].

It is well accepted that the vehicle used in topical formulation may have an influence on the permeation across the skin, because it can modify the diffusion coefficient of the drug or its partitioning into the stratum corneum [16]. Additionally, the vehicle is subject to a “metamorphosis” upon skin application, due to the evaporation of volatile components [17]. Several* in vitro* studies have been done to characterize propranolol HCl skin penetration from solutions [18, 19] or semisolid formulations [15, 20–22]. Other studies were performed using propranolol free base [23] or salts different from the hydrochloride [24]. The effect of pH on skin permeation was characterized by Chantasart and by Casiraghi [19, 21], coming to the expected conclusion that permeability coefficient increases with pH. In one of the cited works [20] it was shown that occlusion can improve propranolol skin permeation from hydroxyl ethyl cellulose gels. As a matter of fact, for the treatment of a localized disease, such as hemangioma, skin retention may be as, or even more, relevant than skin permeation; however, only a few studies [21, 25] have measured propranolol retention in the skin.

The objective of this work was to study* in vitro* propranolol permeation and skin retention after topical application of different semisolid vehicles, with the final aim of posing the basis for the development of new topical formulations, able to produce therapeutic drug level in the skin, avoiding systemic absorption. Propranolol ointments, creams, and gels were prepared and tested on pig skin, an accepted model of human skin. The experiments were performed in infinite dose conditions, to determine propranolol permeation parameters, and in finite dose conditions to determine skin retention. Because IH is treated in infants and it is well known that the skin barrier might be not fully developed, the experiments were performed also on partly stripped skin, to simulate the skin permeability of damaged/immature skin [26, 27].

The formulations chosen were the same or similar to those already tested in clinical trials and shown to be effective. In particular, we chose (i) two hydrophobic ointments to take advantage of the effect of occlusion; (ii) two hydrophobic creams (water-in-oil) to take advantage of a biphasic, occlusive, system; (iii) a hydrophilic gel, often used in clinic.

## 2. Experimental

### 2.1. Chemicals

Propranolol hydrochloride (m.w. 295.807 g/mol, pKa 9.50±0.15, logP octanol/pH 7.4 buffer 1.16±0.57 [18]) was purchased from Sigma Aldrich Italia (Milano, I). White petrolatum, cetostearyl alcohol, liquid paraffin, olive oil, cetyl palmitate, and white beeswax were purchased from ACEF (Fiorenzuola d'Arda, I). Pluronic® F127 (poloxamer 407) was a gift from BASF Italia (Milano, I), Peceol® (gyceryl monoleate) was a gift from Gattefossé (Saint-Priest, F), and Cithrol® GMS (glyceryl monostearate) was a gift from Croda Italiana (Mortara, I).

All other reagents were of analytical grade.

### 2.2. Preparation of Semisolid Formulations

The composition of the semisolid formulations prepared is reported in Table 1.

#### 2.2.1. Preparation of Petroleum Jelly Ointment (Ointment1)

Propranolol hydrochloride 1% (w/w) was levigated with few drops of liquid paraffin and the suspension was incorporated into the white petrolatum using a spatula and a porcelain ointment slab.

#### 2.2.2. Preparation of Ointment2

Ointment2 was prepared according to Deutsches Arzneibuch DAB 2009 (unguentum emulsificans). White petrolatum and cetostearyl alcohol were melt in a water bath at 70°C. After adding propranolol HCl suspended in liquid paraffin and heated at the same temperature under stirring, the mixture was removed from heat and stirred until it began to congeal.

#### 2.2.3. Preparation of Cold Creams

Cold cream (cream1) was prepared according to the Italian Pharmacopoeia FU XII ed. The oily phase (olive oil, cetylpalmitate, white beeswax, and glyceryl monostearate) and the aqueous phase (propranolol HCl dissolved in water) were heated separately in a water bath set at 75°C and then mixed under stirring. Stirring continued until the formulation began to congeal.

A second cold cream (cream2) containing Peceol® instead of olive oil was prepared in the same way.

#### 2.2.4. Preparation of Gel

Poloxamer gel was prepared using the cold method. The polymer (Pluronic® F127) was added under stirring to a water solution of propranolol HCl cooled at 4-5°C. The formulation was left overnight in a refrigerator to ensure complete dissolution.

### 2.3. Tissue Preparation

Pig skin was excised after sacrifice from the outer part of pig ears obtained from a local slaughterhouse (Macello Annoni Spa, Madonna dei Prati, I). Partially compromised skin was prepared by tape stripping full thickness skin 10 times [26], which induced an increase of TEWL of* approx. *50%. Full thickness skin, intact and tape stripped, was frozen after the removal of subcutaneous fat and used within 3 months. Isolated epidermis was prepared, both from intact and tape stripped full thickness skin, by soaking in water at 60°C for 1 min and then peeling off with forceps.

### 2.4. Permeation Studies

Franz-type vertical diffusion cells (Disa, Milano, I), with a diffusional area of 0.6 cm^2^, were used. The epidermis, intact or tape stripped, was mounted between the two halves of the cell, with the stratum corneum facing the donor compartment. The receptor compartment was filled with about 4 ml of degassed NaCl 0.9% (w/v) and kept under magnetic stirring, while in the donor compartment all the formulations were applied in infinite dose conditions (1 g/cm^2^). The receptor compartment was immersed in a thermostatted bath set at 37°C, to guarantee a skin surface temperature of 32°C.

All experiments lasted 24 hours; at predetermined intervals of time, 300 *μ*l of solution was taken from the receptor compartment and replaced immediately with fresh solution.

Blank experiments were also performed in order to check possible tissue interference.

The flux of propranolol across both intact and stripped epidermis (*J*, *μ*g cm^−2^ h^−1^) was calculated as the slope of the regression line at steady state, while the apparent permeability coefficient (*P*_, _cm h^−1^) was calculated at the steady state as

(1) $$\begin{array}{c} P=\frac{J}{C_{D}} \end{array}$$

with* C*_*D*_ (*μ*g ml^−1^) being the concentration of propranolol in the donor formulation. Furthermore,* lag time* (h) was calculated as the intercept the regression line at steady state on time axis.

### 2.5. Skin Retention Studies

Accumulation experiments were conducted using full thickness skin, intact or tape stripped, with the same apparatus described above. The formulations were applied in finite dose conditions (10 mg/cm^2^). After 4 hours of contact, the skin was dismounted from the cell. Excess donor formulation remaining on the skin was wiped 3 times with paper soaked in distilled water followed by 3 times with dry paper. The skin was then cut in correspondence of the permeation area and the epidermis was heat separated from the dermis [28]. Samples were placed in vials and extracted with 1 ml of 0.2% (v/v) H_3_PO_4_ at 30°C overnight. In these conditions, the recovery was higher than 95% for both epidermis and dermis.

### 2.6. HPLC Analysis

Propranolol concentrations in samples were determined using an Agilent HPLC 1260 (Agilent, Santa Clara, CA, USA). Chromatographic separation was achieved using a C18 Novapak column (Waters Italia, Milano, I). The mobile phase consisted of a mixture of 0.2% (v/v) H_3_PO_4_ and acetonitrile (50:50, v/v). The flow rate was 1 ml/min, the injection volume was 100 *μ*l and UV detector was set at 225 nm. The method was validated for sensitivity, recovery, linearity, accuracy, and precision. The LOQ was 0.01 *μ*g/ml.

### 2.7. Statistical Analysis

The significance of differences between values was assessed using one-way ANOVA followed by Bonferroni test (Kaleidagraph 4.5.2 software). Differences were considered statistically significant when p <0.05. In the text, data are reported as mean value ± sd.

## 3. Results and Discussion

Permeation and retention experiments were conducted* in vitro* using pig skin, a well-accepted model of human skin. For the two types of experiments isolated epidermis and full thickness skin were used, in infinite and fine dose conditions, respectively.

Permeation experiments were performed in infinite dose conditions, to determine the relevant permeation parameters. For these experiments, isolated epidermis was used because it simulates better the* in vivo* situation; in fact* in vivo* systemic absorption takes place at the level of upper dermis, where capillaries are located, so the real barrier to permeation is the epidermis [29]. Additionally, when using full thickness skin the dermis might take up water and swell, thus reducing drug diffusion and creating an artifact [30]. Preliminary experiments showed that using full thickness pig skin a permeation lag time of 6-7 h was observed (data not shown).

Propranolol skin retention studies were performed using full thickness pig ear skin and the formulations were applied at finite dose, to mimic more closely the* in vivo* situation. The experiments were limited to 4 h of contact, because this can be a reasonable persistence time of a semisolid formulation on the skin surface. Additionally, it has been shown that the skin acts as a reservoir for propranolol HCl, releasing it also after the formulation is removed [21].

Both sets of experiments were replicated also on partly damaged skin, to evaluate the effect of a damaged barrier on propranolol skin penetration and retention.

Concerning the formulations tested, two ointments, two creams, and one gel were tested. While ointments and creams were taken from clinical studies, the gel was never tested* in vivo*. The gel formulation contains poloxamer 407, a tri-block copolymer of propylene oxide, and ethylene oxide, with thermo-reversible properties, known to form micelles in aqueous solution.

### 3.1. Propranolol Epidermis Permeation

**Figure 1:**
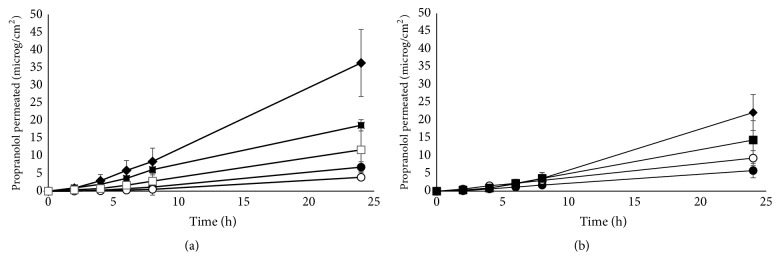
Permeation profiles of propranolol from semisolid formulations (–●– ointment1; –○– ointment2; –■– cream1; –□– cream2; –◆– poloxamer 407 gel) across intact (Panel a) and stripped isolated epidermis (Panel b). Mean values ± sd.

Figure 1(a) reports propranolol permeation profiles obtained from ointments, creams, and gel across full thickness intact pig skin. From the linear portion of the permeation profiles (typically in the interval 4-24 hours) the flux and permeability coefficient were calculated (Table 2).

The permeation of propranolol was markedly higher from poloxamer 407 gel, followed by the creams and the two ointments. Despite the use of isolated epidermis, propranolol permeation showed a significant lag time, of* approx.* 4-6 h.

The value of permeation parameters obtained from poloxamer 407 gel is consistent with literature data obtained using human epidermis and propranolol HCl solutions or gels having comparable concentration and pH [15, 18–21]. This, on one hand, confirms the validity of pig skin as a model of human skin and, on the other hand, suggests no interaction between the micelle-forming polymer and the drug.

The permeation of propranolol from the ointments was similar, but much lower than from the gel. Owing to the composition of the ointments and the use of propranolol HCl, one can assume that the drug was not completely solubilized in the lipophilic vehicles; this means that the concentration of solubilized propranolol is probably lower than the drug loading (1%). For this reason, permeability coefficients were calculated only for the gel and for the two creams, because the drug was not completely solubilized in the ointments and its solubility is unknown.

In their work on propranolol skin penetration and retention, Casiraghi* et al*. [21] reported data from semisolid formulations. The permeation from 1% white petrolatum was below the limit of detection of the analytical method, whereas in our case a measurable flux was obtained. This can be due to the different sensitivity of HPLC method and/or to the method of preparation: in fact, in their case, propranolol HCl was triturated in white paraffin, whereas in our case the drug was firstly levigated with a few drops of liquid paraffin and the obtained suspension was incorporated into the white petrolatum. The different preparation procedure can have had an impact on the physical properties of the drug, such as particle size.

The permeation of propranolol from the creams was intermediate between the gel and the ointments. In this case, propranolol HCl was solubilized in the internal phase of the emulsion (both were w/o emulsions) and the drug had to partition out of the aqueous phase through the lipophilic phase before reaching the skin surface.

In the mentioned work of Casiraghi* et al*., the authors explored the use of lipophilic creams, but the results are not comparable to the ones we obtained, because of the presence of sodium methyl paraben in their formulation, which—as stated by the authors—altered the pH of the formulation.

When partially compromised skin was used (Figure 1(b), Table 2), in general, the flux did not change significantly, with the only exception of ointment2. This is surprising because the permeability barrier of the skin is located in the stratum corneum and its removal should produce, generally speaking, increased permeability, as reported in the literature also for propranolol [19, 21]. However, it should be also kept in mind that the effect of barrier impairment is mediated also by the nature of the vehicle [26]. Additionally, the skin was only partially stripped, up to a 50% increase of TEWL, to simulate the permeability of damaged/immature skin [26, 27], and this has been shown to affect only marginally the permeability of the skin to water [31]. Finally, it has been suggested that, owing to its lipophilicity, the viable epidermis could also contribute as a barrier in propranolol permeation [19, 21].

Overall, the results obtained indicate that a partially impaired barrier, typical of damaged/immature skin, shows the same permeability to propranolol as a healthy barrier.

### 3.2. Propranolol Skin Retention

Propranolol retention in full thickness skin was studied in finite dose conditions, with an application time of 4 hours, which reasonably mimics the real contact time of a semisolid formulation on the skin surface. Finite dose was chosen to simulate more closely what happens* in vivo*, in particular to evaluate the effect of the so called vehicle metamorphosis [17] after application, i.e., evaporation of volatile components.

**Figure 2:**
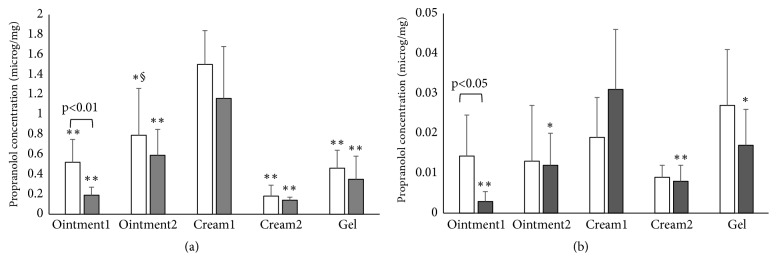
Skin retention of propranolol from semisolid formulations in the epidermis (Panel a) and dermis (Panel b) after 4h of contact. White bars refer to intact skin, grey bars to stripped skin. Mean values ± sd. Statistical differences: with cream1 (*∗* p<0.05; *∗∗* p<0.01); with cream2 (§ p<0.05)). The difference between intact and stripped skin was significant only for ointment1, in both epidermis (p<0.01) and dermis (p<0.05). The formulations were applied at finite dose (10 *μ*g/cm^2^).

Figure 2 reports the amount of propranolol retained in the skin, in both epidermis and dermis, for intact and stripped skin. The data are reported as concentration, i.e., amount of propranolol recovered per unit weight of the tissue, to account for the variability of the different skin specimen. Please note the different Y scale in the two panels. Propranolol was never found in the receptor compartment, in agreement with the long lag time already observed in permeation experiments.

In general, propranolol accumulates more in the epidermis compared to dermis; this is particularly evident in the case of cream1, where epidermis concentration reaches 1.5 *μ*g/mg (corresponding to 13 *μ*g/cm^2^) while dermis concentration is in the order of 0.02 *μ*g/mg (equivalent to 3.4 *μ*g/cm^2^). It is important to underline that dermis retention might be slightly overestimated owing to the absence—*in vitro*—of systemic circulation able to remove the drug present at the level of capillaries; swelling of dermis upon contact with the receptor solution should play a secondary role, due to the short duration of the experiment and to the normalization of the data for the weight of the tissue.

When comparing the different formulations, it appears that epidermis skin retention was maximum for cream1 and minimum for cream2 (p<0.01). Cream2, which differs from cream1 for the oil phase component (olive oil* versus* glyceryl monoleate), was formulated to check the effect of oil phase lipophilicity on propranolol skin retention. Olive oil is highly lipophilic, because it contains mainly triglycerides (in particular triolein), whereas glyceryl monoleate has a higher polar character due to the presence of free hydroxyl groups. The two formulations, of different lipophilicity, originated comparable skin permeation data, but different skin retention data. This can be due to (i) a different “solvent drag effect” in the skin/stratum corneum of the oil phase (olive oil* versus* glyceryl monoleate) [32]; (ii) different formulation metamorphosis of the two creams when applied to the skin surface in finite dose conditions; (iii) a different lag time.

We tried to understand this difference by looking at the permeation parameters (see Table 2); the comparison of lag time for the two creams (Table 2) reveals that cream1 has a faster permeation (lag time 1.03±0.81 h) compared to cream2 (lag time 3.18±1.01 h, p<0.05). The link between lag time and skin retention is not straightforward, but one can imagine that when the steady-state is reached, i.e., the concentration gradient in the skin is linear, the concentration of the permeant is higher, compared to a nonsteady state situation. The skin retention of all monophasic formulations (ointments and gel) was comparable among them, in contrast with the permeation data. Again, the measurement of skin retention in a situation of nonsteady state is probably responsible for the lack of differences (the lag time was in the interval between 3 and 5 hours, see Table 2). The levels of propranolol in the dermis were comparable for the different formulations and this is consistent with the short experimental time with respect to the permeation lag time observed in permeation experiments.

When the formulations were tested on stripped skin, the concentration of propranolol in the epidermis was reduced, although the difference was significant only for ointment1 (p<0.01). If propranolol, in its free base form, forms a reservoir in the stratum corneum, this small reduction could simply reflect the reduced amount of stratum corneum present in the partly tape stripped skin. Propranolol concentration in the dermis decreased (ointment1 and gel), increased (cream1), or remained unchanged (ointment2 and cream2), the difference being significant only for ointment1 (p<0.05). It should be underlined that the data reported in the figure show high variability, in part due also to the data normalization by the weight of the tissue.

**Figure 3:**
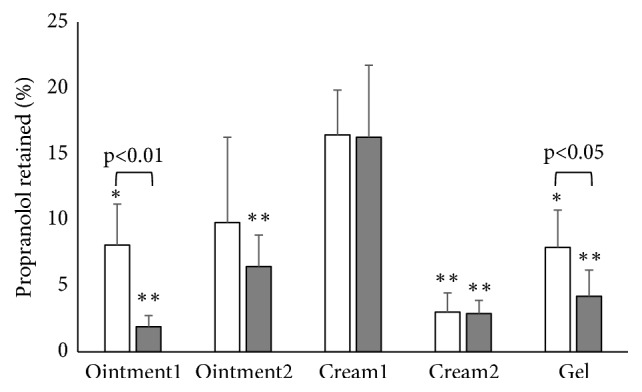
Percentage of propranolol recovered in the skin (epidermis plus dermis) after 4 h of contact. White bars refer to intact skin; grey bars to stripped skin. Mean values±sd. Statistical differences with cream1: *∗* p<0.05; *∗∗* p<0.01. The difference between intact and stripped skin was significant for ointment1 (p<0.01) and for the gel (p<0.05).

Finally, the percentage of propranolol recovered in the skin (epidermis plus dermis), with respect to the amount applied, was calculated and reported in Figure 3. The total amount of propranolol recovered in the skin accounts for 5 to 15% of the amount applied; from the comparison of the different formulations, cream1 is the best performing, significantly higher than ointment1, cream2, and the gel. The data obtained with partly damaged skin confirm this result, making the differences observed among formulations more evident (p<0.01 in all cases). The data obtained on stripped skin is also less variable.

## 4. Conclusion

From the results obtained in the present work it is clear that the permeation of propranolol across isolated epidermis at infinite dose is a poor predictor of its skin retention in finite dose conditions, at least in the time-frame considered. With an application time of 4 h, reasonably close to the permanence time of a semisolid formulation on the skin surface, the best performance (high retention, low skin penetration) was obtained with lipophilic formulations, in particular with a lipophilic cream containing olive oil. The replacement of olive oil with a less lipophilic component, such as glyceryl monoleate, reduced significantly skin retention. Hydrophilic formulations, such as gels, present the problem of a significant absorption, probably leading to systemic side effects, accompanied by a limited skin retention.

When the formulations were applied on partly damaged skin, small differences in permeation and in retention were observed, suggesting that the application of the formulations to immature/partially damaged skin would not pose a toxicological problem.

Overall, the results obtained in the present work pose the basis for the development of new topical formulations, containing propranolol, with better performance and reduced systemic absorption.

## Figures and Tables

**Table 1:** Semisolid formulations composition (% w/w).

Component	Ointment1	Ointment2	Cream1	Cream2	Gel
Propranolol HCl	1.00	1.00	1.00	1.00	1.00
White petrolatum	99.00	34.65	-	-	-
Cetostearyl alcohol	-	29.70	-	-	-
Liquid paraffin	-	34.65	-	-	-
Olive oil	-	-	59.00	-	-
Glyceryl monoleate (Peceol®)	-	-	-	59.00	-
Cetyl palmitate	-	-	7.00	7.00	-
White beeswax	-	-	6.00	6.00	-
Glyceryl monostearate (Cithrol® GMS)	-	-	2.00	2.00	-
Poloxamer 407 (Pluronic® F127)	-	-	-	-	24.75
Water	-	-	25.00	25.00	74.25

**Table 2:** Propranolol permeation parameters across isolated epidermis from porcine skin, intact and partially tape stripped (10 times). All formulation contained 10 mg/ml of drug (mean values±sd).

**Intact skin**			

**Formulation**	**Flux ** **(*μ*g cm** ^**−2**^ ** h** ^**−1**^ **)**	**Permeability coefficient ** **(cm h** ^**−1**^ **)** **∗** **10** ^**5**^	**Lag time** **(h)**

**Ointment1**	0.34±0.07	nc	4.32±0.62
**Ointment2**	0.20±0.08^a^	nc	5.08±1.14
**Cream1**	0.82±0.10	8.16±0.96	1.03±0.8
**Cream2**	0.55±0.31	5.51±3.11	3.18±1.0
**Gel**	1.74±0.73	17.42±7.34	4.16±2.88

**Partially stripped skin**			

**Formulation**	**Flux ** **(*μ*g cm** ^**−2**^ ** h** ^**−1**^ **)**	**Permeability coefficient ** **(cm h** ^**−1**^ **)** **∗** **10** ^**5**^	**Lag time** **(h)**

**Ointment1**	0.26±0.08	nc	1.79±2.11
**Ointment2**	0.39±0.09	nc	1.06±0.82
**Cream1**	0.65±0.32	6.55±3.22	2.79±1.62
**Cream2**	nd	nd	nd
**Gel**	1.13±0.26	11.29±2.57	4.56±1.09

nd: not determined.

nc: not calculable.

Difference between intact and stripped skin: ^a^p < 0.01.

## Data Availability

Data are available upon a direct request to the corresponding author (patrizia.santi@unipr.it).
